# Factors predicting the length of stay in inpatient forensic psychiatric care in Hungary

**DOI:** 10.3389/fpsyt.2025.1582702

**Published:** 2025-05-13

**Authors:** Szabolcs Fekete, Hunor Girasek, Gabor S. Ungvari, Gábor Gazdag

**Affiliations:** ^1^ National Institute of Forensic Psychiatry, Budapest, Hungary; ^2^ Semmelweis University, Doctoral College, Budapest, Hungary; ^3^ Doctoral School of Psychology, ELTE Eötvös Loránd University, Budapest, Hungary; ^4^ Institute of Psychology, ELTE Eötvös Loránd University, Budapest, Hungary; ^5^ Department of Psychiatry and Psychiatric Rehabilitation, Jahn Ferenc South Pest Hospital, Budapest, Hungary; ^6^ Division of Psychiatry, School of Medicine, University of Western Australia, Crawley, WA, Australia; ^7^ Section of Psychiatry, University of Notre Dame, Fremantle, WA, Australia; ^8^ Department of Psychiatry and Psychotherapy, Faculty of Medicine, Semmelweis University, Budapest, Hungary

**Keywords:** length of stay, forensic inpatient care, Hungary, community residential setting, type of criminal offense

## Abstract

**Introduction:**

In recent years, the length of stay (LoS) in forensic psychiatric inpatient institutions has been increasing worldwide. Although an excessive LoS constitutes a human rights violation, the underlying reasons and associated factors have not been fully established, and differences between countries and regions have led to limited conclusive information.

**Methods:**

This retrospective study used data from a nationally representative sample of 301 adult patients who were admitted to the National Institute of Forensic Psychiatry, Hungary’s only forensic inpatient psychiatric institution, following a court-issued compulsory treatment order for reason of insanity during 2000–2015 to investigate the demographic, clinical, and criminal offense-related factors associated with inpatient LoS.

**Results:**

Among the variables found to be significant in univariate analyses, a multiple regression model with bootstrapping confirmed significant associations of LoS with the type and method of the index criminal offense, community residential setting into which the patient was released, age and gender (all p < 0.05). However, the regression model accounted for only 34.1% of the observed variance in LoS, suggesting the presence of additional unexplored factors that may lead to excessive LoS.

**Conclusion:**

The Hungarian forensic psychiatric system should implement a more evidence-based method for determining the LoS for inpatient care.

## Introduction

The factors influencing the duration of inpatient treatment are among the most important recent research areas in forensic psychiatry (1). An excessive length of stay (LoS) in forensic psychiatric institutions constitutes a violation of human rights, places undue burden on forensic health care systems and incurs substantial costs (2–4). Recent decades have seen an increasing trend of forensic inpatient stays lasting for months or even years (5, 6), such that the duration of such an inpatient stay may be even longer than the duration of incarceration for offenders convicted on the same charges without a psychiatric diagnosis (3, 7). In some countries (e.g., Hungary), the legal framework allows an offender to receive inpatient treatment in forensic psychiatric institutions beyond the maximum length of a prison sentence. This scenario carries a risk of an excessive LoS in forensic institutions, which may violate fundamental human rights (7). In recent years, such LoS-related violations have led to increasing criticism of inpatient forensic psychiatric treatment (2, 8). Although the reasons for the increase in LoS are not yet clear, one probable factor is societal demand for strong and decisive measures against offenders with mental disorders to minimize their risk of re-offending (9).

To understand the increase in LoS, the associated demographic and clinical factors must first be identified when seeking an optimal therapeutic approach to reduce an unnecessarily long LoS and the rational use of financial resources (7, 9). The duration of forensic hospitalization differs significantly between countries and even between regions within a country due to significant geographic variations in treatment standards, the provision of inpatient forensic care services and the legal framework (2, 10, 11). In a study of 12 European countries, forensic inpatients were found to have an average LoS of less than 3.5 years in seven countries and more than 7 years in the others, and the between-country differences were large (1–10 years) (11). Furthermore, European countries use different definitions of criminal responsibility and mental illness (10, 11). In some countries (e.g., Republic of Ireland, Latvia, Lithuania), forensic psychiatric care is only available to patients with severe psychiatric disorders (e.g., psychotic disorders), while in others (e.g. Belgium, Germany, Italy, Poland, Serbia), patients with personality disorders can be admitted to inpatient forensic psychiatric institutions (9, 10). A comprehensive literature review found that the duration of forensic hospitalization is influenced mainly by the legal framework and broader socio-cultural environment (12).

The clinical and socio-demographic variables influencing LoS in inpatient forensic psychiatric settings can be classified as (a) personal demographics, including educational and vocational qualifications and work and family history; (b) clinical data, including medical conditions and mental health; and (c) criminal history (4).

Factors known to be associated with longer LoS include male gender (4, 13, 14), older age at admission (4, 15, 16), single marital status (4, 17, 18), lower education level (16–19), lower IQ (17), lack of housing at the time of discharge from forensic inpatient treatment (4, 15) and unemployment prior to the index admission (16–18, 20). However, some studies have concluded that sociodemographic variables have little or no effect on the LoS (1, 6, 8, 21).

Among clinical factors, diagnosis of a schizophrenia spectrum disorder was found to have the most significant impact on the LoS (1, 4, 6, 7, 13, 14, 22–26). Some studies have found that alcohol or substance abuse also may increase the LoS (24, 26), but other studies have found no or even the opposite effect (1, 8, 13, 23). Studies have also identified correlations between an increased LoS and the duration of mental illness (1), previous admission to a psychiatric institution (4) and a family history of psychiatric disorders (7).

Furthermore, most studies have concluded that the severity of the index crime leading to forensic inpatient admission is an important predictor of the LoS (2, 8): the more serious the index crime, the longer the stay (1, 14–19, 23, 24, 27, 28). Researchers have identified particularly strong correlations of a long LoS with committed/attempted homicide (6, 7) and other violent crimes (23–26). A long LoS has also been associated with crimes committed against known persons (17) or committed earlier in life (2, 29).

Only a few studies examined treatment or institutional behavior characteristics with respect to LoS (6). Factors associated with longer LoS include absconding from the institution during treatment (2, 4, 9), treatment-resistant psychotic symptoms (7), aggressive attacks against staff (2, 4), lack of cooperation, and lack of insight about mental illness (2).

The aims of this study were to identify the demographic, clinical and crime-related factors influencing the LoS. The patient sample comprised forensic psychiatric patients who had been found not guilty by reason of insanity and sentenced to receive compulsory treatment in the only forensic inpatient psychiatric facility in Hungary. According to the Hungarian Criminal Code (C/2012. Criminal Code), persons could be sentenced to compulsory forensic psychiatric treatment if (1) they committed a violent crime or caused a public danger, and (2) due to their pathological mental state they are not guilty by reason of insanity and there is a risk that they will commit a similar crime, and (3) if their crime is punishable for more than one year of imprisonment.

The institute, where these patients must serve their sentences is the National Institute of Forensic Psychiatry (NIFP), which is under the direction of the Minister of Internal Affairs, and its operation is supervised by the Hungarian Prison Service. This setting provided a unique opportunity to study a nationally representative sample of severely mentally ill offenders requiring admission. Relevant data from Hungary are limited, and this study would enable the establishment of a comprehensive national database.

Another aim of this study was to explore community reintegration pathways in the context of the LoS of forensic patients. In Hungary, and possibly elsewhere, the scarcity of social aftercare in the forensic psychiatric system is a major problem. However, this topic has not been extensively studied in any country.

## Methods

### Sample

The NIFP was established [1] to assess and treat psychiatrically ill offenders who were not found guilty of crimes by reason of insanity and [2] to provide short-term psychiatric treatment for prisoners who become mentally ill while serving their sentences. The NIFP is part of the Hungarian Prison Service and located within the premises of the Budapest Medium and Maximum Security Prison. It comprises three buildings with 311 beds across one acute psychiatric, one female and two male psychiatric rehabilitation units. All Hungarian offenders with severe mental illness who require admission are managed in one facility, providing a unique opportunity to establish a comprehensive database that covers the entire country.

This study focuses *only* on inpatients who were found not guilty by reason of insanity and underwent court-ordered treatment. In Hungary, the Court orders such offenders to undergo psychiatric treatment in lieu of a prison sentence for an indefinite period, followed by a case review every six months. The Court then decides whether to maintain or terminate treatment based on the opinion of the attending consultant psychiatrist or Chief of Service of the NIFP and an independent forensic psychiatric expert. The latter professional recommends discontinuation of inpatient treatment if the patients’ condition has sufficiently improved or their treatment adherence is sufficiently stable to enable a shift to outpatient treatment through public psychiatric services while living with their families or in supervised community residential settings. In Hungary, no forensic outpatient treatment is available. On the day when the court responsible for reviewing the necessity of compulsory forensic inpatient treatment decides about the discharge, patients must be released to the care of the public community psychiatric services while residing either at their homes or moving in to a community residential setting.

For this retrospective study, the medical records of all patients who received a court-issued compulsory treatment order for reason of insanity and were discharged from the NIFP between January 1, 2000 and December 31, 2015 were reviewed following a pilot project by two authors (GG and SF) to determine the scope of data extraction from notes based on an earlier local study (29). The following demographic data were collected: age, gender, residential and marital status, education level, pre-offense employment and number of children and guardianship. The following clinical variables were extracted: diagnoses according to the International Classification of Diseases, 10^th^ Revision (ICD-10), family history of psychiatric disorders, history of alcohol and substance use, and alcohol and/or substance use immediately prior to committing the index criminal offense. The following index criminal offense variables were collected: type of offense, method of offense, relation to the victim, offense under the influence of alcohol or drugs, history of violence, recidivism in terms of offending, and/or forensic psychiatric treatment.

Sexual offenders can be treated in NIFP only if they are not guilty by reason of insanity. Otherwise they serve their sentence in prison in a separate unit. In our sample there were only 10 patients who committed sexual offenses. Due to statistical reasons, this subgroup was merged with those who were convicted for an assault.

Treatment-related factors can also influence LoS in forensic inpatient facilities, but due to the retrospective nature of our study, a few potentially important factors affecting LoS, such as type of drugs, their doses, polypharmacy, the length of pharmacotherapy, structured assessment of treatment response, and concomitant psychotherapy and rehabilitative efforts, were not consistently available, therefore they could not be included in the analysis. A prospective study can target these issues, but, unfortunately, conducting a prospective study involving forensic inpatients is legally forbidden in Hungary (CLIV/1997. Health Act, 161 § (3),: “Detained persons or persons under military service cannot be subjects of research even if they consent.”).

### Ethics approval

The study was approved by the Scientific and Research Ethics Committee of the Medical Research Council of Hungary (No: 51124-1/2016/EKU). The research was conducted according to the ethical principles of the Declaration of Helsinki issued by the World Medical Association (30).

### Statistical analysis

Statistical analyses were performed using the SPSS software package (Version 30.0; IBM Corporation, Armonk, NY, USA) (31). Effect size measures were calculated using ROPstat statistical software on the R platform (32). Descriptive data are reported as means and standard deviations, and non-parametric statistical data are reported as medians. The normality of numerical variables was determined using the Kolmogorov–Lilliefors test and skewness and kurtosis. As the distributions of these variables diverged significantly from normal distribution (p < 0.01), non-parametric tests were used for further analyses, including the Mann–Whitney U test and Kruskal–Wallis test. Spearman’s rho correlations were used to evaluate continuous variables.

When building the regression model, first the Forward and Backward selection methods were used, including all variables of study. Subsequently, the final model was performed using the Enter method with a bootstrap procedure (Number of samples 999, seed: 2000000, Bias corrected accelerated) to avoid normality violation. The sample size was set to 999 instead of 1000 based on Wilcox (33). For the regression models, the categorical variables were binarized to be included in the analysis.

Due to the non-parametric nature of the tests, rank Cohen’s d was used as the measure. Rather than using means and standard deviations as for Cohen’s d, rank Cohen’s d is calculated using mean ranks and standard deviations of the ranks (34). Effect size indicators were interpreted according to Cohen (35).

## Results

The study sample consisted of 301 inpatients (48 women, 16%; 253 men, 84%) who were released from NIFP between January 1, 2000 and December 31, 2015. The mean age of the sample at the time of admission was 40.23 ± 14.59 years, and the mean LoS was 68.05 ± 47.59 months (median: 53 months). The patients’ sociodemographic data are shown in **Table 1**. The index offense and forensic, clinical and historical descriptive data are provided in **Table 2**.

**Table 1:** Sociodemographic data of the sample.

Variables	Number	Percentage	Mean	Range	SD
Total number of patients	301	100%			
Length of stay (months)			68.05	2–285	47.59
The period when patients were released					
2000-2005	87	28.9%			
2006-2010	131	43.5%			
2011-2015	83	27.6%			
Demographic data					
Age, years			40.23	18–87	14.59
Female	48	16%			
Male	253	84%			
Highest educational level					
Special education	21	7%			
Less than eight years of primary school	23	7.6%			
Eight years of primary school completed	107	35.5%			
Secondary or technical school	104	34.6%			
High school	21	7%			
Higher education	22	7.3%			
Employment status at the time of offense					
Disability pensioner	144	47.8%			
Employed	51	16.9%			
Retired	24	8%			
Unemployed	78	25.9%			
Student	4	1.3%			
Residence status before the index offense					
Village	87	28.9%			
Homeless	7	2.3%			
Town/city	191	63.5%			
Supervised community facility	16	5.3%			
Marital status					
Single	158	52.5%			
Married	94	31.2%			
Divorced	49	16.3%			
Number of children					
None	158	52.5%			
One	64	21.3%			
Two	44	14.6%			
Three or more	35	11.6%			
Guardianship					
No	244	81.1%			
Yes	57	18.9%			
IQ	179	59.5%	94.06	40–134	18.64

**Table 2:** Index offense, forensic, clinical and historical descriptive data of the sample.

Variables	Number	Percentage
Type of index offense		
Homicide/attempted homicide	146	48%
Assault/sexual offense	137	46%
Property crimes	18	6%
Method of index offense		
Physical force	82	27%
Weapon/other object	219	73%
Relation to victim		
Unknown person	84	27.9%
Close relative	137	45.5%
Acquaintance	68	22.6%
Could not be determined	12	4%
Offense under the influence of alcohol		
No	247	82.1%
Yes	54	17.9%
Residential setting in which patients were released		
Supervised community facility	136	45.2%
Family	31	10.3%
Own property/home	113	37.5%
Deceased in custody	20	6.6%
Forensic history		
No	189	62.8%
Yes	111	36.9%
History of violence		
No	194	64.5%
Yes	106	35.2%
Diagnosis at admission (ICD-10)		
F00–F09 Organic, including symptomatic, mental disorders	50	16.6%
F20–F29 Schizophrenia, schizotypal and delusional disorders	232	77.1%
F30–F39 Mood (affective) disorders	7	2.3%
F70–F79 Mental retardation	12	4%
Psychiatric history		
No	83	27.6%
Yes	218	72.4%
Diagnosis during previous psychiatric treatment		
F00–F09 Organic, including symptomatic, mental disorders	3	1%
F10–F19 Mental and behavioral disorders due to psychoactive substance use	19	6.3%
F20–F29 Schizophrenia, schizotypal and delusional disorders	123	40.9%
F30–F39 Mood (affective) disorders	15	5%
F40–F48 Neurotic, stress-related and somatoform disorders	11	3.7%
F60–F69 Disorders of adult personality and behavior	13	4.3%
F70–F79 Mental retardation	15	5%
Family history of psychiatric disorders		
No	171	56.8%
Yes	130	43.2%

### Correlations of socio-demographic, clinical and forensic factors with LoS

Gender was found to be significantly associated with LoS (Z = –1.989; p = 0.047), with men having a significantly longer median LoS than women (59.00 *vs*. 46.00 months); the effect size was intermediate (Cohen’s d = –0.315). A weak but significant negative correlation was found between age and LoS (rs = –0.142; p = 0.013). Employment status also was significantly associated with LoS (H(4) = 15.823; p = 0.003), although the effect size was small (η^2^ = 0.053). Pairwise comparisons showed that patients who were unemployed or receiving a disability pension had significantly longer LoS (p < 0.05) than others. A significant association was found between LoS and marital status (H(2) = 7.239; p = 0.027), with single patients having a longer median LoS than married and divorced patients (63.50 *vs*. 46.00 *vs*. 56.00 months); however, the effect size was small (η^2^ = 0.024). The number of children also was significantly associated with a longer LoS (H(3) = 8.309; p = 0.040), although the effect size was small (η^2^ = 0.028). Specifically, patients with no children had a significantly longer median LoS than those with 1 or 3 children (62.00 *vs*. 47.00 *vs*. 51.00 months, p < 0.05); no significant differences were observed between groups with other numbers of children. A weak but significant negative relationship was observed between IQ and LoS (rs = –0.231; p = 0.002). As IQ showed a weak relationship with LoS and only 59.5% of the total sample had IQ data available, this variable was not included in further analyses. No significant relationships were found between LoS and other sociodemographic variables such as education level, type of residence, or presence of guardianship or the discharge period. The statistical results for demographic variables are shown in **Table 3**.

**Table 3:** Univariate correlation of sociodemographic variables with LoS.

	Median (months)	Mean Rank	Statistics Effect size
Sex			
Female	46.00	128.09	**Z = -1.989;** **p = 0.047** **Cohen’s d = -0.315**
Male	59.00	155.35	
The period when patients were released			
2000-2005	61.00	161.16	H(2) = 4.440; p = 0.109 η^2^ = 0.015
2006-2010	47.00	138.98	
2011-2015	56.00	159.32	
Highest educational status achieved			
Special education for the intellectually impaired	84.00	165.86	H(5) = 10.134; p = 0.072 η^2^ = 0.034
Less than eight years of primary school	61.00	170.70	
Eight years of primary school completed	56.00	151.45	
Secondary or technical school	56.00	154.37	
High school	40.00	109.17	
Higher education	41.50	117.70	
Employment status at the time of offense			
Disability pensioner	63.50	161.55	**H(4) = 15.823; p = 0.003** **η^2^ = 0.053**
Employed	44.00	127.79	
Retired	39.00	112.04	
Unemployed	59.50	163.02	
Student	25.00	66.38	
Residence status before the index offense			
Village	61.00	155.97	H(3) = 1.227; P = 0.746 η^2^ = 0.004
Homeless	47.00	143.71	
Town/city	52.00	150.73	
Supervised community facility	44.00	130.44	
Marital status			
Single	63.50	163.00	**H(2) = 7.239;** **P = 0.027** **η^2^ = 0.024**
Married	46.00	132.71	
Divorced	56.00	147.41	
Number of children			
None	62.00	163.65	**H(3) = 8.309;** **P = 0.040** **η^2^ = 0.028**
One	47.00	135.01	
Two	51.00	148.31	
Three or more	51.00	126.53	
Guardianship			
No	56.00	152.49	Z = 0.613; P = 0.540 Cohen’s d = 0.090
Yes	47.00	144.64	
Residential setting in which patients were released			
Supervised community facility	79.50	190.80	**H(3) = 56.918;** **P < 0.001** **η^2^ = 0.190**
Family	43.00	136.08	
Own property/home	41.00	108.93	
Deceased	45.00	133.70	

Significant results are highlighted in bold in the Table.

A significant relationship was found between the type of index offense and LoS (H(2) = 12.201; p = 0.002), with a small effect size (η^2^ = 0.041). Specifically, patients who committed homicide/attempted homicide had a significantly longer median LoS than those admitted for assault or crimes against property (63.00 vs. 46.00 vs. 49.00 months; p = 0.002). Additionally, patients who committed a crime with a weapon or object had a significantly longer median LoS than those who used physical force alone (59.00 vs. 46.50, Z = –2.144; p = 0.032), although the effect size was small (Cohen’s d = –0.279).

A significant association was found between LoS and the type of community residential setting into which patients were released (H(3) = 56.918; p < 0.001), with a large effect size (η^2^ = 0.190). In pairwise comparisons, patients discharged to a supervised community facility had a significantly longer median LoS (79.50 months) than those who were released to a family home or their own property or died while in the NIFP (43.00 vs. 41.00 vs. 45.00 months); no other significant between-group differences were observed. No significant associations of LoS were observed with relation to the victim, offense under the influence of alcohol, forensic history, or history of violence. The statistical results regarding the characteristics of the index offense are shown in **Table 4**.

**Table 4:** Univariate correlation of offense-related variables with LoS.

	Median	Mean Rank	Statistic Effect size
Type of index offense			
Homicide/attempted homicide	63.00	169.00	**H(2) = 12.201;** **P = 0.002** **η^2^ = 0.041**
Assault	46.00	134.73	
Property crime	49.00	128.86	
Method of index offense			
Physical force	46.50	133.43	**Z = -2.144;** **p = 0.032** **Cohen’s d = -0.279**
Weapon/Other object	59.00	157.58	
Relation to victim			
Unknown person	51.00	146.70	H(3) = 2.037; p = 0.565 η^2^ = 0.007
Close relative	59.00	157.71	
Acquaintance	53.50	146.93	
Could not be determined	46.50	127.58	
Offense under the influence of alcohol			
No	55.00	152.86	Z = 0.795; p = 0.427 Cohen’s d = 0.119
Yes	46.50	142.47	
Forensic history			
No	53.00	149.57	Z = -0.241; p = 0.809 Cohen’s d = -0.029
Yes	59.00	152.08	
History of violence			
No	54.00	148.99	Z = -0.408; p = 0.683 Cohen’s d = -0.049
Yes	53.00	153.26	

Significant results are highlighted in bold in the Table.

Regarding clinical factors, only a previous psychiatric diagnosis had a significant association with LoS (H(6) = 14.326; p = 0.026), with a medium effect size (η^2^ = 0.072). Compared with schizophrenia, the ICD-10 diagnostic groups of schizotypal and delusional disorders (61.00 months), mood (affective) disorders (74.00 months) and organic mental disorders (105.00 months), and neurotic, stress-related and somatoform disorders (35.00 months) and a previous diagnosis of mental retardation (36.00 months) were all associated with a significantly shorter LoS (p < 0.05). Furthermore, patients with a history of psychoactive substance use had a significantly shorter median LoS (46.00 months) than those with organic mental disorders (105 months; p = 0.05). Pairwise comparisons revealed no other significant differences between previous diagnoses. Furthermore, LoS was not significantly associated with clinical variables such as the diagnosis at admission and personal or family history of psychiatric disorders. The statistical results of clinical variables are presented in **Table 5**.

**Table 5:** Effect of clinical variables on LoS.

	Median	Mean Rank	Statistic Effect size
Diagnosis at admission			
F00–F09 Organic, including symptomatic, mental disorders	50.00	140.35	H(3) = 0.916; p = 0.821 η^2^ = 0.003
F20–F29 Schizophrenia, schizotypal and delusional disorders	53.50	152.93	
F30–F39 Mood (affective) disorders	55.00	153.93	
F70–F79 Mental retardation	78.00	156.38	
Psychiatric history			
No	48.00	144.79	Z = -0.764; p = 0.445 Cohen’s d = -0.098
Yes	56.00	153.36	
Diagnosis during previous psychiatric treatment			
F00–F09 Organic, including symptomatic, mental disorders	105.00	161.67	**H(6) = 14.326;** **p = 0.026** **η^2^ = 0.072**
F10–F19 Mental and behavioral disorders due to psychoactive substance use	46.00	91.61	
F20–F29 Schizophrenia, schizotypal and delusional disorders	61.00	104.43	
F30–F39 Mood (affective) disorders	74.00	115.43	
F40–F48 Neurotic, stress-related and somatoform disorders	35.00	69.64	
F60–F69 Disorders of adult personality and behavior	67.00	104.35	
F70–F79 Mental retardation	36.00	65.00	
Family history of psychiatric disorders			
No	56.00	149.63	Z = -0.314; p = 0.753 Cohen’s d = -0.037
Yes	52.00	152.81	

Significant results are highlighted in bold in the Table.

In order to find out which factors have a predictive effect on LoS, multiple linear regressions were performed. For building the regression model, first the Forward and Backward stepwise multiple linear regressions were performed, including all the variables studied in the study. The final Forward selection model (**Table 6**) had 5 variables explaining 32.5% of LoS (F(5, 274) = 26.351, p < 0.001). The final Backward selection model (**Table 7**) had 11 variables explaining 36.6% of LoS (F(11, 268) = 14.052, p < 0.001). The results of the two regression models were then tested in one model using the Enter method. The variables that did not show a significant effect in the final Enter method model (**Table 8**) were removed, with the resulting model explaining 34.1% of the LoS (F(8, 272) = 17.589, p < 0.001). The model also showed a significant effect after the bootsrap procedure in the following variables: supervised community facility – residential setting to which patients were released (b = 44.392 [34.694, 54.723], p = 0.001), homicide/attempted homicide – Type of index offense (b = 22.933 [12.522, 32.993], p = 0.001), Age (b = -0.688 [-1.018, -0.356], p = 0.001), F70–F79 Mental retardation diagnosis during previous psychiatric treatment (b = -35.665 [-59.442, -15.038] p = 0.003), male sex (b = 13.821 [2.832, 25.922], p = 0.018), supervised community facility – residence status before the index offense (b = -21.229, [-37.588, -4.662], p = 0.013), F70–F79 Mental retardation diagnosis at admission (ICD-10) (b = 34.047 [1.364, 65.696], p = 0.041), and physical force – method of index offense (b = -10.208 [-20.850, -0.350], p = 0.036).

**Table 6:** Multiple regression analysis using Forward selection method of variables with a significant effect on LoS.

Forward Model	Unstandardized Coefficients		Standardized Coefficients	t	Sig.
	B	Std. Error	Beta		
(Constant)	54.605	9.760		5.595	0.000
Supervised community facility (Residential setting in which patients were released)	44.681	4.752	0.474	9.403	0.000
Homicide/attempted homicide (Type of index offense)	25.739	4.683	0.274	5.496	0.000
Age	-0.731	0.161	-0.229	-4.533	0.000
F70–F79 Mental retardation (Diagnosis during previous psychiatric treatment)	-30.714	10.521	-0.147	-2.919	0.004
Male (Sex)	13.349	6.593	0.102	2.025	0.044

**Table 7:** Multiple regression analysis using backward selection method of variables with a significant effect on LoS.

Backward Model	Unstandardized Coefficients		Standardized Coefficients	t	Sig.
	B	Std. Error	Beta		
(Constant)	81.935				
Student (Employment status at the time of offense)	-42.190	19.992	-0.107	-2.110	0.036
Supervised community facility (Residence status before the index offense)	-22.678	11.031	-0.109	-2.056	0.041
Supervised community facility (Residential setting in which patients were released)	43.818	4.700	0.465	9.323	0.000
Assault (Type of index offense)	-21.760	5.129	-0.231	-4.242	0.000
Property crimes (Type of index offense)	-26.173	10.158	-0.130	-2.576	0.011
F70–F79 Mental retardation (Diagnosis at admission (ICD-10))	38.151	14.418	0.158	2.646	0.009
F00–F09 Organic, including symptomatic, mental disorders (Diagnosis during previous psychiatric treatment)	40.226	22.457	0.088	1.791	0.074
F70–F79 Mental retardation (Diagnosis during previous psychiatric treatment)	-37.218	11.511	-0.179	-3.233	0.001
Male (Sex)	15.113	6.487	0.115	2.330	0.021
Physical force (Method of index offense)	-11.320	5.825	-0.107	-1.943	0.05
Age	-0.760	0.164	-0.238	-4.635	0.000

**Table 8:** Final multiple regression analysis using enter method of variables with a significant effect on LoS.

Enter Model (Bootstrap)	B	Bootstrap				
		Bias	Std. Error	Sig. (2-tailed)	BCa 95% Confidence Interval	
					Lower	Upper
(Constant)	56.524	-0.202	9.781	0.001	35.776	74.634
Supervised community facility (Residential setting in which patients were released)	44.392	0.236	4.901	0.001	34.694	54.723
Homicide/attempted homicide (Type of index offense)	22.933	-0.041	5.095	0.001	12.522	32.993
Age	-0.688	-0.003	0.168	0.001	-1.018	-0.356
F70–F79 Mental retardation (Diagnosis during previous psychiatric treatment)	-35.665	0.290	10.356	0.003	-59.442	-15.038
Male (Sex)	13.821	0.301	5.648	0.018	2.832	25.922
Supervised community facility (Residence status before the index offense)	-21.229	-0.257	8.845	0.013	-37.588	-4.662
F70–F79 Mental retardation (Diagnosis at admission (ICD-10))	34.047	-0.473	17.112	0.041	1.364	65.696
Physical force (Method of index offense)	-10.208	0.187	4.707	0.036	-20.850	-0.350

## Discussion

To the best of our knowledge, this is the first study to explore the factors influencing LoS in the national Hungarian inpatient forensic psychiatric service. To date, little has been known about LoS in such services Eastern or Central European countries [e.g (1, 4)]. Although the mean LoS, 68.05 ± 47.59 months, initially appears to be extremely long, it is average for Europe. A much longer LoS has been reported in the Netherlands, Germany, Scotland, England, Wales and Ireland, with a shorter LoS in Lithuania, Latvia, Poland and Slovenia (11).

Several studies have attempted to identify the sociodemographic factors that influence LoS in forensic institutions, with conflicting results. Although a recent systematic review found strong evidence that gender is not correlated with LoS (6), the present study found a moderate correlation between male gender and higher LoS, consistent with other studies (4, 13, 36, 37). This correlation remained significant in a multivariable analysis, possibly because male patients are perceived as being more dangerous than female patients.

Of all the variables investigated in this study, the correlation of the community residential setting (previous supervised community facility placement negatively, while the waitlist for release to supervised community placement positively predicted LoS) had the largest effect size with LoS. Possibly, the chronic shortage of supervised community accommodations in Hungary (38) caused patients qualified to enter such facilities to wait twice as long in the NIFP than other patients in the sample, which would be an ethically unacceptable outcome. No significant correlations were found between LoS and other sociodemographic variables.

Factors associated with the index offense were found to be strong predictors of LoS (17, 39, 40). Homicide or attempted homicide was one of the strongest predictors in the current study, confirming this well-established correlation (6). The use of a weapon or other object was also significantly correlated with LoS in this study. Specifically, committing a crime with a weapon or other object usually results in more serious injury to the victim, which has also been correlated with a longer LoS (2, 16, 24).

The majority (77%) of patients in the sample were diagnosed with a psychotic disorder (ICD-10 diagnostic codes F20–29) in the NIFP. The small number of patients with other diagnoses might explain why psychotic diagnosis was not found to be associated with LoS in this study, in contrast to the literature (4, 23, 24, 41). In a univariate analysis, a psychiatric history (72.4% of the sample) was significantly correlated with LoS, but this correlation disappeared in the multivariable analysis. Case-by-case analysis revealed that a significant number of patients had received different diagnoses in the NIFP than during previous psychiatric treatment episodes. For example, the number of patients diagnosed with a psychotic disorder increased from 123 to 232. Misdiagnosis of psychotic disorders during previous treatment for adjustment, affective, or personality disorders or drug-induced behavioral disturbances may explain this difference. Misdiagnosis of mental retardation can also explain the findings of the final regression model since its previous diagnosis was correlated with shorter, while its diagnosis at admission to the NIFP with longer LoS.

Sociodemographic, clinical and offense-related variables explained only 34.1% of the variance in multiple linear regressions, implying that the main determinants of LoS remain hidden. This finding is rather alarming because the majority of factors predicting LoS are unknown thereby preventing the forensic psychiatric services to implement corrective measure to shorten LoS. Therefore, it is imperative to broaden the current clinical standard of forensic psychiatric assessment and documentation as well as involving more input from allied mental health professionals, relatives and the patients themselves.

The retrospective nature of this study may have precluded the inclusion of several variables with potential effects on LoS, such as the patients’ early social and developmental history, the adequacy of pharmacological and psycho-social treatment at the NIFP, and an estimate of societal tolerance toward violent offenders. Beyond professional aspects, decision-making forensic psychiatrists and judges may choose to err on the side of caution to protect the community from re-offenders, which may also have a significant effect on LoS. The necessity of continuation of forensic psychiatric treatment is evaluated every 6 months for all patients under compulsory inpatient treatment order in NIFP. The treating psychiatrist gives a recommendation for the continuation of treatment or for discharge of the patients. Then, an independent forensic psychiatric expert examines the patients, reviews their documentation, and gives a recommendation to the judge who makes the final decision. If the judge terminates the compulsory treatment order, the patient must be released within 24 hours from NIFP. However, if there is no adequate community place available, either a protective, safe family home or in a residential community setting, the judge has no choice but to extend the inpatient compulsory treatment. This is an entirely unsatisfactory situation and warrants urgent actions to expand the availability of community residential places in Hungary.

### Strengths and limitations of the study

This is the first study involving a comprehensive evaluation of the factors influencing LoS in the only forensic inpatient psychiatric facility in Hungary. The results of this study are therefore representative of Hungarian forensic psychiatric care nationwide. Unlike in Hungary, inpatient forensic psychiatric care in most European countries is not centralized in one place, but provided in a number of institutes, that may have different levels of security, different protocols, different scales for diagnostic or risk evaluation, and different treatment strategies. Having a full picture about the inpatient forensic psychiatric system of a given European country thus can be complicated. The strength of the current study is the use of data collected in a single place where all Hungarian forensic inpatients are treated, thus eliminating these difficulties in data interpretation. Another strength is that the results point out the deficiencies in social aftercare of forensic psychiatric patients affect LoS; previous studies have paid less attention to this potential factor regarding LoS (2, 15, 18).

The majority of the limitations of this study stem from its retrospective nature. While every attempt was made to extract as much information from patients’ files as possible, the quality of the data limited the validity of the results. For example, data on psychopathological symptoms were omitted due to the large number of confounding factors (e.g., missing information, time elapsed since the offense, emergency treatment before detailed forensic examination). A further limitation is the lack of structured diagnostic interviews or symptom assessment scale and psychological test results apart from IQ data for 179 patients. Data on pharmacological and psychosocial treatment, rehabilitative efforts and the use of risk and functional assessment scales were not included in the study and should be targeted in further studies.

## Conclusion

In this retrospective study, 34.1% of the variance in LoS in the Hungarian forensic inpatient psychiatric service was explained by male gender, age, a conviction for murder or attempted murder, use of a weapon or other objects and the community residential setting in which patients were released. Our results clearly indicate the presence of additional factors not accounted for in the available data.

Unjustifiably extended LoS and long waiting times for supervised community residential facilities may violate patients’ human rights. The Hungarian forensic psychiatric system should make further efforts toward implementing a more evidence-based method for determining LoS in inpatient care.

## Data Availability

The raw data supporting the conclusions of this article will be made available by the authors, without undue reservation.
